# The Dutch Data Warehouse, a multicenter and full-admission electronic health records database for critically ill COVID-19 patients

**DOI:** 10.1186/s13054-021-03733-z

**Published:** 2021-08-23

**Authors:** Lucas M. Fleuren, Tariq A. Dam, Michele Tonutti, Daan P. de Bruin, Robbert C. A. Lalisang, Diederik Gommers, Olaf L. Cremer, Rob J. Bosman, Sander Rigter, Evert-Jan Wils, Tim Frenzel, Dave A. Dongelmans, Remko de Jong, Marco Peters, Marlijn J. A. Kamps, Dharmanand Ramnarain, Ralph Nowitzky, Fleur G. C. A. Nooteboom, Wouter de Ruijter, Louise C. Urlings-Strop, Ellen G. M. Smit, D. Jannet Mehagnoul-Schipper, Tom Dormans, Cornelis P. C. de Jager, Stefaan H. A. Hendriks, Sefanja Achterberg, Evelien Oostdijk, Auke C. Reidinga, Barbara Festen-Spanjer, Gert B. Brunnekreef, Alexander D. Cornet, Walter van den Tempel, Age D. Boelens, Peter Koetsier, Judith Lens, Harald J. Faber, A. Karakus, Robert Entjes, Paul de Jong, Thijs C. D. Rettig, Sesmu Arbous, Sebastiaan J. J. Vonk, Mattia Fornasa, Tomas Machado, Taco Houwert, Hidde Hovenkamp, Roberto Noorduijn-Londono, Davide Quintarelli, Martijn G. Scholtemeijer, Aletta A. de Beer, Giovanni Cina, Martijn Beudel, Willem E. Herter, Armand R. J. Girbes, Mark Hoogendoorn, Patrick J. Thoral, Paul W. G. Elbers

**Affiliations:** 1 Laboratory for Critical Care Computational Intelligence, Department of Intensive Care Medicine, Amsterdam Medical Data Science, Amsterdam UMC, Vrije Universiteit, Amsterdam, The Netherlands; 2 Pacmed, Amsterdam, The Netherlands; 3 Department of Intensive Care, Erasmus Medical Center, Rotterdam, The Netherlands; 4 Intensive Care, UMC Utrecht, Utrecht, The Netherlands; 5 ICU, OLVG, Amsterdam, The Netherlands; 6 Department of Anesthesiology and Intensive Care, St. Antonius Hospital, Nieuwegein, The Netherlands; 7 Department of Intensive Care, Franciscus Gasthuis & Vlietland, Rotterdam, The Netherlands; 8 Department of Intensive Care Medicine, Radboud University Medical Center, Nijmegen, The Netherlands; 9 Department of Intensive Care Medicine, Amsterdam UMC, Amsterdam, The Netherlands; 10 Intensive Care, Bovenij Ziekenhuis, Amsterdam, The Netherlands; 11 Intensive Care, Canisius Wilhelmina Ziekenhuis, Nijmegen, The Netherlands; 12 Intensive Care, Catharina Ziekenhuis Eindhoven, Eindhoven, The Netherlands; 13 Department of Intensive Care, ETZ Tilburg, Tilburg, The Netherlands; 14 Intensive Care, HagaZiekenhuis, Den Haag, The Netherlands; 15 Intensive Care, Laurentius Ziekenhuis, Roermond, The Netherlands; 16 Department of Intensive Care Medicine, Northwest Clinics, Alkmaar, The Netherlands; 17 Intensive Care, Reinier de Graaf Gasthuis, Delft, The Netherlands; 18 Intensive Care, Spaarne Gasthuis, Haarlem en Hoofddorp, The Netherlands; 19 Intensive Care, VieCuri Medisch Centrum, Venlo, The Netherlands; 20 Intensive Care, Zuyderland MC, Heerlen, The Netherlands; 21 Department of Intensive Care, Jeroen Bosch Ziekenhuis, Den Bosch, The Netherlands; 22 Intensive Care, Albert Schweitzerziekenhuis, Dordrecht, The Netherlands; 23 ICU, Haaglanden Medisch Centrum, Den Haag, The Netherlands; 24 ICU, Maasstad Ziekenhuis Rotterdam, Rotterdam, The Netherlands; 25 ICU, SEH, BWC, Martiniziekenhuis, Groningen, The Netherlands; 26 Intensive Care, Ziekenhuis Gelderse Vallei, Ede, The Netherlands; 27 Department of Intensive Care, Ziekenhuisgroep Twente, Almelo, The Netherlands; 28 Department of Intensive Care, Medisch Spectrum Twente, Enschede, The Netherlands; 29 Department of Intensive Care, Ikazia Ziekenhuis Rotterdam, Rotterdam, The Netherlands; 30 Anesthesiology, Antonius Ziekenhuis Sneek, Sneek, The Netherlands; 31 Intensive Care, Medisch Centrum Leeuwarden, Leeuwarden, The Netherlands; 32 ICU, ICU, IJsselland Ziekenhuis, Capelle aan den IJssel, The Netherlands; 33 ICU, WZA, Assen, The Netherlands; 34 Department of Intensive Care, Diakonessenhuis Hospital, Utrecht, The Netherlands; 35 Department of Intensive Care, Admiraal De Ruyter Ziekenhuis, Goes, The Netherlands; 36 Department of Anesthesia and Intensive Care, Slingeland Ziekenhuis, Doetinchem, The Netherlands; 37 Department of Intensive Care, Amphia Ziekenhuis, Breda, The Netherlands; 38 Department of Intensive Care, LUMC, Leiden, The Netherlands; 39 Department of Neurology, Amsterdam UMC, Universiteit van Amsterdam, Amsterdam, The Netherlands; 40 Quantitative Data Analytics Group, Department of Computer Science, Faculty of Science, Vrjie Universiteit, Amsterdam, The Netherlands

**Keywords:** Database, Big data, COVID-19, Data sharing

## Abstract

**Background:**

The Coronavirus disease 2019 (COVID-19) pandemic has underlined the urgent need for reliable, multicenter, and full-admission intensive care data to advance our understanding of the course of the disease and investigate potential treatment strategies. In this study, we present the Dutch Data Warehouse (DDW), the first multicenter electronic health record (EHR) database with full-admission data from critically ill COVID-19 patients.

**Methods:**

A nation-wide data sharing collaboration was launched at the beginning of the pandemic in March 2020. All hospitals in the Netherlands were asked to participate and share pseudonymized EHR data from adult critically ill COVID-19 patients. Data included patient demographics, clinical observations, administered medication, laboratory determinations, and data from vital sign monitors and life support devices. Data sharing agreements were signed with participating hospitals before any data transfers took place. Data were extracted from the local EHRs with prespecified queries and combined into a staging dataset through an extract–transform–load (ETL) pipeline. In the consecutive processing pipeline, data were mapped to a common concept vocabulary and enriched with derived concepts. Data validation was a continuous process throughout the project. All participating hospitals have access to the DDW. Within legal and ethical boundaries, data are available to clinicians and researchers.

**Results:**

Out of the 81 intensive care units in the Netherlands, 66 participated in the collaboration, 47 have signed the data sharing agreement, and 35 have shared their data. Data from 25 hospitals have passed through the ETL and processing pipeline. Currently, 3464 patients are included in the DDW, both from wave 1 and wave 2 in the Netherlands. More than 200 million clinical data points are available. Overall ICU mortality was 24.4%. Respiratory and hemodynamic parameters were most frequently measured throughout a patient's stay. For each patient, all administered medication and their daily fluid balance were available. Missing data are reported for each descriptive.

**Conclusions:**

In this study, we show that EHR data from critically ill COVID-19 patients may be lawfully collected and can be combined into a data warehouse. These initiatives are indispensable to advance medical data science in the field of intensive care medicine.

**Supplementary Information:**

The online version contains supplementary material available at 10.1186/s13054-021-03733-z.

## Introduction

The Corona virus disease 2019 (COVID-19) pandemic has placed an unprecedented burden on intensive care units around the world. Many intensive care units still face high death rates, and the number of critically ill patients still exceeds available intensive care unit (ICU) beds in some areas [1]. More than ever before, COVID-19 has shown the need for concerted research efforts among the intensive care community to understand the course of severe COVID-19 disease, to identify potential treatment strategies and to guide resource allocation.

Research with routinely collected electronic health record (EHR) data has increasingly gained interest in the ICU over the last decade [2]. There has been a widespread transition toward EHR systems, enabling the routine capture of individual patient data throughout ICU admission [3]. Moreover, several individual hospitals have extracted these EHR data and converted them into critical care datasets available for research, including the Medical Information Mart for Intensive Care (MIMIC) [4], AmsterdamUMCdb [5], and HiRID [6]. These datasets have laid the groundwork for working with EHR data and have advanced medical data science in the field of critical care.

However, rather than single-center data alone, the COVID-19 pandemic has underlined the need for accurate and verifiable multicenter data [7, 8]. The novelty of COVID-19 and absence of treatment guidelines resulted in practice variation between centers, emphasizing the limits of single-center research and the need for multicenter research into effective treatment strategies [9]. Furthermore, medical transfers, different levels of care, and care practice differences between hospitals hamper the extrapolation of single-center data. Patient demographics, for example, have been shown to differ considerably between centers [10]. Multicenter data are therefore crucial, but assembling data from multiple centers yields major challenges.

We initiated a large-scale data sharing collaboration in the Netherlands that resulted in the Dutch Data Warehouse (DDW), a complete-admission and multicenter database with EHR data from critically ill COVID-19 patients. The DDW was designed with an interdisciplinary team of legal advisors, privacy officers, data engineers, IT-professionals, data scientists, statisticians, and clinicians. This paper presents a full report on the first stable version of the database and addresses the major challenges in the construction of the DDW. Given the crisis, a brief overview of the preliminary dataset was published as a letter [11]. In the present report, we expand on the methodology underlying the DDW and show the patient population currently included.

## Methods

**Fig. 1:**
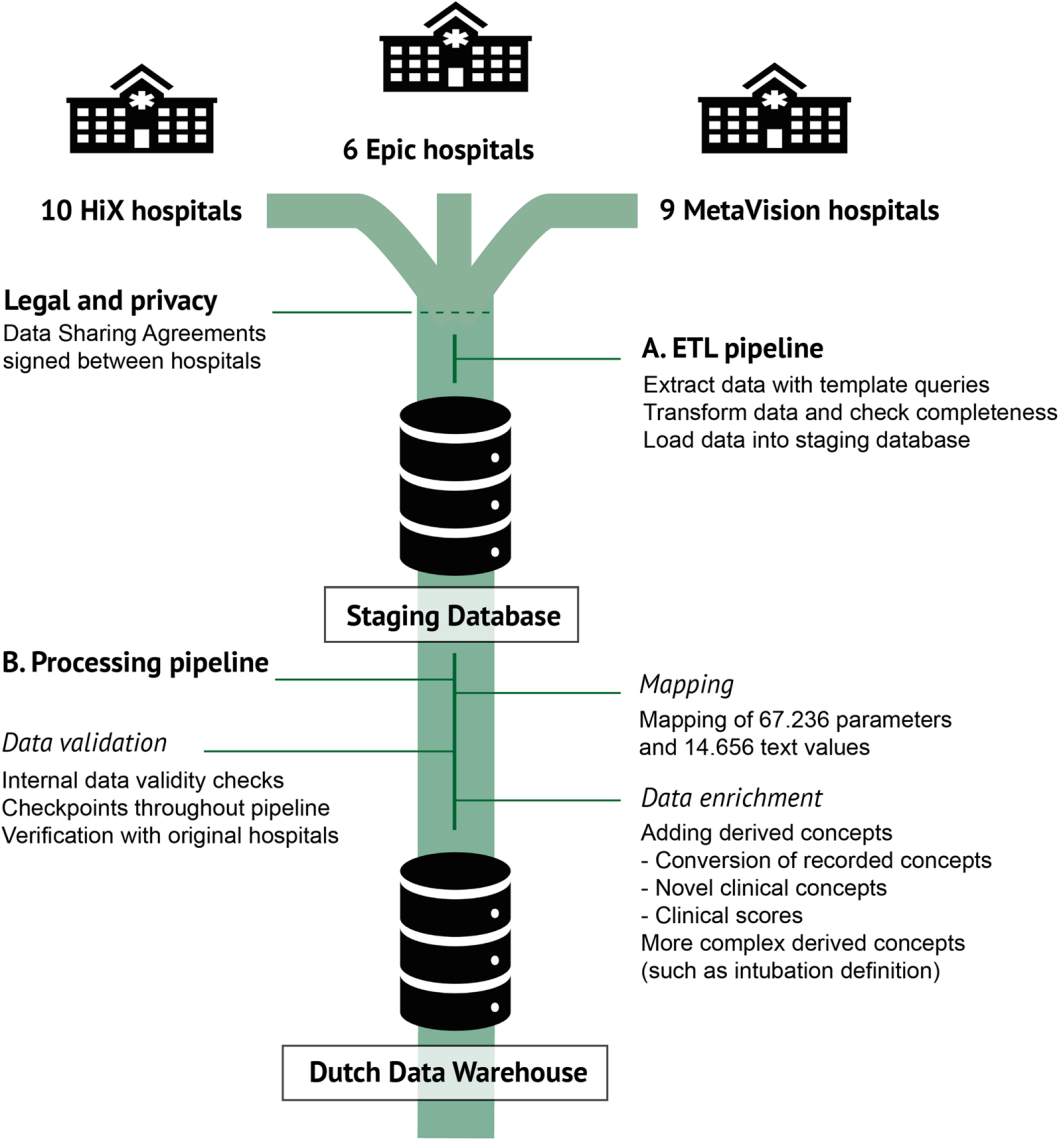
Overview of the Dutch Data Warehouse pipeline. Overview of the collaboration to realize the Dutch Data Warehouse. *EHR* electronic health record, *ETL* extract–transform–load

The data sharing collaborative was started at the beginning of the COVID-19 crisis in the Netherlands in March 2020. All hospitals in the Netherlands with an intensive care unit were approached to participate. Per hospital, an intensivist and IT-professional served as contacts for local study approval, data expertise, and data extraction. All hospitals that participated have access to the cumulative dataset for research purposes. The process of obtaining legal approval and the extract–transform–load (ETL) pipeline, as well as the data mapping, data enrichment, and data validation process are described in detail. An overview of the project can be found in Fig. 1.

### Legal and privacy

In close collaboration with data protection officers (DPO), health care lawyers, and intensivists, we drafted a data sharing agreement (DSA) and a multidisciplinary report on the lawful collection of EHR data during the COVID-19 crisis. Under the General Data Protection Regulation (GDPR) and Dutch law, data subjects are required to give explicit consent for the processing of their data. We argued, however, that during the COVID-19 crisis asking consent could not be reasonably expected from health care workers due to (a) the large number of expected patients and associated time burden in an already overstrained health care system, (b) the danger of spreading or contracting the virus upon contact with patients or their families, and (c) the poor clinical condition of many patients in the intensive care. Consent was therefore not only impractical, but often infeasible. In addition, alternative forms of data collection to construct a database of this size were unavailable and selection bias would have ensued in case of failed consents.

As under non-crisis circumstances, COVID-19 data necessary for scientific purposes may be gathered when researchers “provide for suitable and specific measures to safeguard the fundamental rights and interests of the data subject” (GDPR, Article 9, paragraph j) [12]. Therefore, we (a) pseudonymized data in the providing hospital, (b) informed patients through media and local hospital outlets about the possibility to opt out, and (c) signed data sharing agreements regulating privacy of patients. The study proposal and documentation were reviewed and approved by the institutional review board of Amsterdam UMC location VUmc prior to study onset. Data sharing agreements were approved locally in each hospital before data transfers took place. The DSA has been added to the Additional files 1 and 2. All institutional review board documentation is available upon request from the corresponding author.

### Extract–transform–load pipeline

In collaboration with local IT-experts, template Structured Query Language (SQL) queries were written to automatically extract EHR data from each of the major EHR systems in the Netherlands: MetaVision (iMDsoft, Tel Aviv, Israel), HiX (ChipSoft, Amsterdam, The Netherlands), and Epic (Epic Systems, Verona, WA, United States). Intensive care COVID-19 patients were labelled locally by the participating hospitals. All adult patients with laboratory-confirmed COVID-19 or a Reporting and Data System (CO-RADS) score with clinical suspicion compatible with the diagnosis were labeled for inclusion (13).

The extracted data included demographics, clinical observations manually entered by the clinical team, administered medication, laboratory determinations, and data from vital sign monitors and life support devices such as mechanical ventilators, renal replacement devices and extracorporeal life support devices. Clinical notes, radiology reports and images, pathology and microbiology data were not extracted due to the additional complexity of these data and potential privacy implications. We included Dutch national registry data on patient comorbidities since these data are unsystematically recorded in the EHR and are frequently part of clinical notes [13].

IT experts from the participating hospitals adjusted the structured queries to local system configurations and performed the data extraction and pseudonymisation. Pseudonymisation was performed using a Secure Hash Algorithm (SHA-256). Data were stored in CSV format and shared with end-to-end encryption. Data extractions were performed upon request depending on the number of newly admitted patients. Upon receiving the data transfers, tables from the different EHR systems were restructured and data were combined into a staging database. A first data validation step was performed checking tables for completeness of columns, missing data, headers, and delimiters. This process was repeated per hospital to ensure completeness of data. After the staging database, data went through the data processing pipeline to be mapped, enriched, further validated and restructured to facilitate research.

### Data mapping

One of the major challenges in combining multicenter EHR data is to find corresponding parameters between hospitals. No mandated set of recorded parameters exists for ICUs in the Netherlands, nor is there a standardized nomenclature for parameters, which results in between-hospital differences on several levels. First, parameter names may differ between hospitals and may include abbreviations, generating a plethora of unique parameters. In addition, certain parameters may be recorded in one hospital, but not in another. For example, not all hospitals record Richmond Agitation and Sedation Scales (RASS). Moreover, the level of parameter detail may differ between hospitals. One hospital may distinguish between alanine transferase (ALAT) measured in blood versus ALAT measured in other body fluids. Lastly, varying units between centers further hampers finding corresponding parameters. These between-hospital differences greatly complicate the combination of multicenter EHR data.

Through a process called mapping, parameters from different hospitals are linked to a concept from a predefined vocabulary. Although international vocabularies such as Logical Observation Identifiers Names and Codes (LOINC) and Systematized Nomenclature of Medicine Clinical Terms (SNOMED CT) exist [14–16], no widespread mapping tooling is available and existing vocabularies may not yet be complete for the intensive care unit [17]. Considering the urgency of the COVID-19 pandemic, we therefore created our own vocabulary of 942 clinically relevant parameters. We incorporated all 5.456 medications included in the Anatomical Therapeutic Chemical (ATC) classifications from the World Health Organization Collaborating Center for Drugs Statistics Methodology [18]. Most, but not all hospitals specified ATC codes for administered medication. Medications without an ATC code were mapped manually. Finally, we created a separate vocabulary of categories for 54 categorical concepts such as heart rhythm. These vocabularies included prespecified concepts for these categories, such as atrial fibrillation, ventricular tachycardia, and so on in the case of heart rhythm.

The received parameters were manually mapped per hospital to the predefined concept vocabulary. In order to facilitate the mapping process, the median, interquartile ranges, number of measurements, min, max, number and percentage of unique patients with the parameter, unit, and the most frequent value were calculated per parameter and exported to Google sheets for the mapping. Consequently, the concepts were aggregated into higher level concepts by the clinical team. For example, temperatures measured in the bladder and esophagus were both aggregated into the higher-level temperature concept. Both the detailed as well as the aggregated mappings are available in the DDW. Next, units were checked for each parameter and adjusted where necessary. Lastly, all mappings were independently reviewed by an intensive care clinician and discussed with the original hospital in case of uncertainty about the mapping. An overview of the most frequent concepts in the DDW can be found in Table 1.

**Table 1:** Most frequent parameters in the Dutch Data Warehouse by number of observations

Category	Parameter name	Parameter subname	Obs	Pat	Hosp.
Respiratory	fio2	fio2 unspecified	3,242,962	385	7
Respiratory	fio2	fio2 set	6,143,804	2927	25
Respiratory	fio2	fio2 optiflow	633	70	1
Respiratory	fio2	fio2 hiflow	7223	266	5
Respiratory	fio2	fio2 measured	2,095,235	1809	20
Respiratory	fio2	fio2 niv	707	65	2
Respiratory	Vent mode	Vent mode	10,614,561	2970	25
Respiratory	Vent mode	Vent mode invasive	15,580	756	11
Respiratory	Vent mode	Vent mode machine	8755	396	4
Respiratory	Vent mode	Vent mode manual	644	105	5
Respiratory	Vent mode	Vent mode noninvasive	3630	315	6
Respiratory	Peep	Peep set	5,976,874	2389	24
Respiratory	Peep	Peep unspecified	20,479	116	2
Respiratory	Peep	Peep measured	3,786,729	2432	22
Hemodynamics	Heart rate	Heart rate ECG	827	179	3
Hemodynamics	Heart rate	Heart rate unspecified	7,095,759	2920	25
Hemodynamics	Heart rate	Heart rate monitor	836,776	740	5
Respiratory	o2 saturation	Saturation o2 peripheral monitor	1,677,619	654	4
Respiratory	o2 saturation	Saturation o2 peripheral	5,447,816	3086	23
Hemodynamics	Arterial blood pressure mean	Arterial bp mean	6,538,822	3284	24
Respiratory	Lung compliance static	Lung compliance static	1,281,683	317	6
Respiratory	Lung compliance static	Lung compliance static adjusted	4,739,724	2515	25
Hemodynamics	Arterial blood pressure systolic	Arterial bp systolic	5,901,827	3286	24
Hemodynamics	Arterial blood pressure diastolic	Arterial bp diastolic	5,896,330	3285	24
Respiratory	Pressure above peep	Pressure support set	240,321	23	1
Respiratory	Pressure above peep	Inspiratory pressure above peep	5,427,631	2429	24
Respiratory	Driving pressure	Driving pressure	4,851,963	2519	25
Respiratory	Driving pressure	Driving pressure adjusted	746,687	1662	22
Respiratory	Tidal volume	Tidal volume expiratory	3,694,052	2546	22
Respiratory	Tidal volume	Tidal volume expiratory measured	1,413,883	122	3
Respiratory	Tidal volume	Tidal volume expiratory unspecified	19,056	109	1
Respiratory	Tidal volume	Tidal volume measured	26,262	32	1
Respiratory	Tidal volume	Tidal volume set	409,944	1383	22
Respiratory	Peak pressure	Peak pressure measured	156,117	18	1
Respiratory	Peak pressure	Peak inspiratory pressure	5,364,952	2687	25
Respiratory	Respiratory rate spontaneous	Respiratory rate measured spontaneous	4,799,514	934	9
Respiratory	Respiratory rate spontaneous	Respiratory rate measured ventilator spontaneous	489,793	1270	13
Respiratory	Tidal volume per kg	Tidal volume per kg	5,240,012	2683	25
Respiratory	Minute volume	Minute volume unspecified	791,194	287	6
Respiratory	Minute volume	Minute volume set	9414	239	5
Respiratory	Minute volume	Minute volume expiratory	4,408,260	2488	22
Respiratory	Respiratory rate measured ventilator	Respiratory rate measured ventilator	5,010,244	2408	23
Respiratory	Mean pressure	Mean airway pressure	4,590,042	2291	22
Respiratory	End tidal co2	End tidal co2 measured	4,193,165	2597	24
Respiratory	Flow trigger set	Flow trigger set	3,531,317	1247	20

*Obs* number of observations, *Pat* number of patients with at least one observation, *Hosp* number of hospitals with at least one observation, *FiO2* fraction of inspired O2, *vent mode* ventilation mode

### Data enrichment

Because several medical concepts are insufficiently stored in the EHR, we added derived concepts to the DDW based on clinical expertise. These concepts included the conversion of recorded concepts, the addition of novel clinical concepts, and the calculation of clinical scores. The conversion of concepts ensured that concepts were added to the database when they could be derived from other available concepts. For example, respiratory system compliance can be calculated when tidal volume and driving pressure are available [19]. Secondly, clinical concepts that have been described in the literature were added to the DDW and included ventilatory ratio [20], physiologic dead space [21], and mechanical power [22]. These derived concepts can be found in Table 2 and included specific algorithms per concept to ensure the correct selection of underlying parameters. Lastly, clinical scores such as the Sequential Organ Failure Assessment (SOFA) score [23] and the Acute Physiology and Chronic Health Evaluation II (APACHE II) score [24] were calculated from the data per calendar day for each patient and can be found in Additional file 3: Table S1.

**Table 2:** Derived parameters in the Dutch Data Warehouse

Parameter	Calculation	Tolerance	Comments
Basophils percentage	Basophils divided by leukocytes	1 h	
Body mass index	Height divided by weight squared	/	A measurement is added for each measurement of weight, using the latest available height for the patient
Driving pressure	Plateau pressure minus peep	8 h/1 h	
Driving pressure	Tidal volume divided by lung compliance static	1 h	
Eosinophils percentage	Eosinophils divided by leukocytes	1 h	
Glasgow coma scale total	Glasgow coma scale eye plus Glasgow coma scale motor plus Glasgow coma scale verbal	1 h	
Inspiratory expiratory ratio	Inspiratory time divided by expiratory time	1 h	
Lung compliance dynamic	Tidal volume divided by (peak pressure minus peep)	1 h	
Lung compliance static	Tidal volume divided by (plateau pressure minus peep)	1 h/8 h/1 h	
Lung compliance static	Tidal volume divided by pressure above peep	1 h	
Lymphocytes percentage	Lymphocytes divided by leukocytes	1 h	
Mechanical power per kg	Mechanical power divided by ideal body weight	/	Ideal body weight is calculated from the patient's gender and height
Mechanical power	(0.098 × respiratory rate measured ventilator times tidal volume times (peak pressure minus (driving pressure × 0.5)) × 0.001	1 h	
Mechanical power	(0.098 × respiratory rate measured ventilator times tidal volume times (peak pressure plus peep) × 0.001	1 h	
Minute volume derived	(tidal volume times respiratory rate measured) × 0.001	1 h	
Monocytes percentage	Monocytes divided by leukocytes	1 h	
Neutrophils percentage	Neutrophils divided by leukocytes	1 h	
Pao2 over fio2	po2 arterial divided by fio2	8 h/1 h	Measurements of ‘fio2’ within 8 h *before* ‘po2’ are given priority over those measured within 1 h afterwards
Pco2 minus end tidal co2	pco2 arterial minus end tidal co2	8 h	Measurements of ‘end tidal co2’ within 1 h *before* `pco2` are given priority over those measured afterwards
Physiological dead space	pco2 minus end tidal co2 minus pco2 arterial	8 h	Measurements of ‘pco2 arterial’ within 8 h *before* ‘pco2 minus end tidal co2’ are given priority over those measured within 8 h afterwards
Plateau pressure	Driving pressure minus peep	8 h/1 h	
Po2 unspecified over fio2	po2 unspecified divided by fio2	8 h/1 h	Measurements of ‘fio2’ within 8 h *before* ‘po2’ are given priority over those measured within 1 h afterwards
Rapid shallow breathing index	(Respiratory rate set divided by tidal volume) × 1000	1 h	
Respiratory rate difference	Respiratory rate measured ventilator minus respiratory rate set	1 h	
Tidal volume per kg	Tidal volume divided by ideal body weight	/	Ideal body weight is calculated from the patient's gender and height
Ureum over creatinine	Ureum minus creatinine	1 h	
Ventilatory ratio	(minute volume times pco2 arterial) divided by (ideal body weight × 100 × 37.5)	1 h	Ideal body weight is calculated from the patient's gender and height

Overview of all derived parameters and their calculation in the data warehouse. The tolerance gives the hours that two measurements may be apart. In case multiple observations are found in that time window, the slash indicates the hierarchy. i.e., 1 h/8 h/1 h means observations are included 1 h before, otherwise 8 h before, or lastly 1 h after the other measurement

In addition to the derived concepts, some concepts required more complex derivation algorithms. Notably, patient in- and extubation times may not be easily or reliably available in EHR data, or result from multiple data columns. Therefore, we developed an algorithm that determines the start and end of intubation episodes based on other concepts. The overview of this algorithm has been published previously [11].

### Data validation

Data validation and quality control were integrated throughout the project. The internal validity of the data was safeguarded by incorporating data that were validated by the clinical team during routine care, comparing calculated clinical scores against the manually recorded benchmarking scores from national registry data, and by data verification checks with the original hospital. In addition, several checkpoints ensured accurate processing of the data throughout the ETL and data processing pipeline. First, patient tables, headers, and column data were checked for completeness in the ETL pipeline. Secondly, parameter mappings were checked by an intensive care clinician and were therefore independently performed by two clinicians. Next, value distribution plots were continuously generated as part of the processing pipeline. These plots show the distribution of all parameters from all hospitals that were mapped to a certain concept and easily identify aberrant mappings. For all concepts, medically impossible cutoff values were determined by the clinical domain experts. Finally, demographics and any inconsistencies in the distributions or mapping were validated with their original hospital.

### Data and code availability

The pipelines were constructed in Python 3 (Python Software Foundation). The resulting DDW is stored on a remote server. An application programming interface (API) was developed to facilitate data access. Access to the server is regulated to comply with the data sharing agreements. All hospitals have access to the data. External researchers can get access to all data in collaboration with any of the participating hospitals. The list of collaborators is available in the co-author list and in the declarations section. The collaborators may be contacted directly, through the corresponding author, or through the contact information on Amsterdammedicaldatascience.nl [25]. Research questions have to be in the line with the reason for data collection as outlined in the DSA; the investigation of the ICU course of COVID-19 or its potential treatments. In addition, researchers have to sign a code of conduct before getting access to the data. Data access is granted by Amsterdam UMC; compliance with the DSA is the responsibility of the researcher and hospital accessing the data. A repository to process the data warehouse, including more information on table structures and data content, is available on Gitlab. Anyone can get access to the repository by contacting the corresponding author.

## Results

The data sharing collaboration was initiated in March 2020. Out of 81 hospitals with an intensive care unit in the Netherlands, 66 hospitals currently participate in the project (7 hospitals did not have the IT infrastructure or resources to carry out the data extraction, 1 hospital did not treat COVID-19 patients, and 7 did not want to participate or did not respond), 47 have signed the data sharing agreement and 35 have shared their data. The time to get approval and extract data ranged between less than 1 month and 6 months between hospitals. So far, data from 25 hospitals have passed through the ETL and data processing pipelines and are currently included in the DDW. These hospitals amount to a total of 3463 patients, both from wave 1 and wave 2 in the Netherlands. From these patients, more than 200 million clinical data points are available.

### Parameter mapping

The mapping process of the received parameters resulted in a large mapping structure between all hospitals and EHR systems. From the staging database, 67,236 parameters (32,570 parameters from EPIC, 19,492 from Hix, and 15,174 parameters from MetaVision) were mapped to the common vocabulary. Next, 14,656 text parameters were mapped to categorical concepts. Part of these mappings were aggregated into 289 higher level concept names. The final list of the most frequent concepts and their clinical categories can be found in Table 1.

### Data tables

**Fig. 2:**
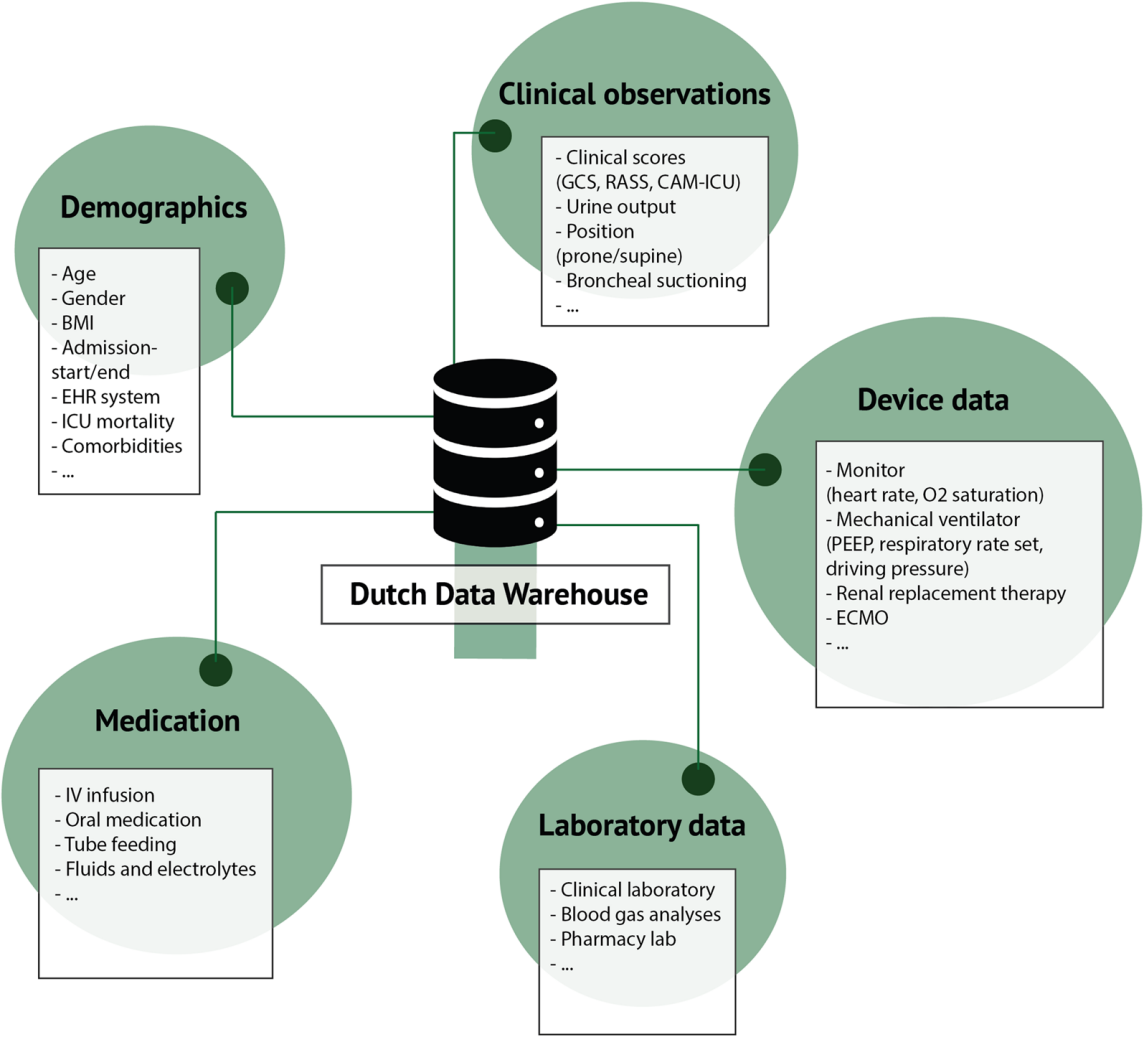
Overview of the Dutch Data Warehouse content. Overview of the data domains in the Dutch Data Warehouse. Examples of data are given per domain. *EHR* electronic health record, *BMI* body mass index, *GCS* Glasgow Coma Scale, *RASS* Richmond agitation and sedation scale, *CAM-ICU* confusion assessment method for the ICU, *PEEP* positive end-expiratory pressure, *ECMO* extracorporeal membrane oxygenation, *IV* intravenous

Figure 2 gives an overview of the included data in the DDW. Table 1 lists the most frequent concepts found in the DDW with the number of total measurements, and the number of patients and number of hospitals with at least one measurement available for that concept. The data are available in separate tables and include a patients table with demographics and admission details; a single-timestamp table with all observations and measurements recorded at a single point in time; a range measurements table that contains parameters with a start and an end timestamp such as urine output, fluid output, and body position; a medications table with start times, end times, and dosing information; a diagnosis table with ICD-10 codes when available; a parameters table with the summary of all parameters currently included in the DDW; an intubations table with the start and end of invasive mechanical ventilation; a comorbidities table; and an outcomes table.

### Clinical characteristics of patients

Table 3 describes the COVID-19 patients currently included in the DDW. The first patient was admitted on February 20, 2020, while the last patient was admitted on March 2, 2021. The median age was 64.0 (IQR 56.0, 72.0), and the majority of patients were male with a median BMI of 27.3 (IQR 24.3, 30.7). Overall ICU mortality was 24.4%.

**Table 3:** Overview of patients in the Dutch Data Warehouse

Patient characteristics	Dutch Data Warehouse cohort	*n*
Age, years (median, IQR)	64.0 (56.0, 72.0)	3459
Body Mass Index, kg/m^2^ (median, IQR)	27.3 (24.3, 30.7)	849
Gender male, (*n*, %)	2498 (72.3)	3457
Transfers^a^, (*n*, %)	367 (10.6)	3463
Referrals^a^, (*n*, %)	784 (26.5)	2963
ICU mortality, (*n*, %)	707 (24.4)	2900
ICU or hospital mortality^b^, (*n*, %)	853 (29.4)	2900
Still admitted^c^, (*n*, %)	196 (5.7)	3463
ICU length of stay, days (median, IQR)	7.1 (2.2, 16.0)	3306
Intubated, (*n*, %)	2644 (75.6)	3496
Length of intubation, days (median, IQR)	7.7 (2.9, 14.3)	2644
CRRT, (*n*, %)	427 (12.2)	3496

Patients admitted in the participating hospitals between March 2020 and March 2021

Overview of characteristics of patients currently included in the Dutch Data Warehouse

*IQR* interquartile range, *CRRT* continuous renal replacement therapy

^a^Patients are transferred from the ICU to other hospitals. Referrals are transfers received from other hospitals

^b^Hospital mortality was not available for 5 hospitals because of different ICU and hospital EHRs

^c^Patients still admitted at the time of data extraction

Importantly, the DDW includes data throughout the ICU admission. The most common parameters were respiratory parameters, notably the fraction of inspired oxygen, the ventilation mode, and the positive end expiratory pressure. These parameters are measured and stored directly by the mechanical ventilator. Similarly, hemodynamic parameters that are automatically recorded and stored are most prevalent, including heart rate and blood pressure. Lastly, fluid balance and all administered medications are available for each patient. Missing data are reported in a separate column for each descriptive.

## Discussion

In this study, we present the Dutch Data Warehouse, a large multicenter database with electronic health record data collected throughout the ICU admission of critically ill COVID-19 patients in the Netherlands. Currently, the DDW contains 3463 patients with over 200 million data points. The first stable version has been released and is available to researchers within ethical and legal boundaries.

The intensive care unit is a natural habitat for large data sharing collaboratives, as much data are collected through routine monitoring, life support devices, and by the clinical team. Although many publicly available single-center datasets have advanced our understanding of electronic health record data [4–6], multicenter data are crucial to enhance generalizability of results and account for between-center differences. The most important aspects of multicenter EHR data sharing include the legal framework, between-hospital concept mapping, and data preparation. Despite the complexity and volume of parameters received, we describe the legal basis for collecting these data under European privacy laws and show that these data can technically be combined into a data warehouse suitable for research.

The DDW has been used both as a research database and to create reports per hospital to compare local practices. The high granularity of the data, the wide variety of clinical parameters, and the availability of the data throughout the ICU stay make the database especially suitable for research. Clinical questions in a wide variety of areas relating to COVID-19 may be answered with the data, such as ventilation strategies, the timing and effects of proning, and the occurrence of superinfections. Apart from hard clinical endpoints such as mortality or length of stay, the DDW also allows for the investigation of intermediate clinical endpoints, such as line infections or improvements in P/F ratios. In addition to research, the dataset was used to create reports for hospitals to discuss and learn from treatment variation. These reports were created upon request and discussed confidentially with the participating hospitals.

For any medical data science project, and in particular projects throughout the COVID-19 pandemic, understanding and verifying the underlying data is crucial to interpret results. Reports have expressed worries about the quality of research conducted throughout the pandemic [26, 27]. The call for accurate, timely and reliable research data is larger than ever before. Only then, research can be replicated and checked by the scientific community. Undoubtedly, there will be mistakes and missing data in the Dutch Data Warehouse. Despite rigorous data preparation and validation, we believe that transparency of data and data sharing is key to continuously and collaboratively improve the dataset. Importantly, knowledge of intensive care medicine is indispensable when reviewing and evaluating the data, and thus, the involvement of critical care clinicians is paramount. With this report, we hope to encourage clinicians and researchers to get involved in data sharing collaborations. Moreover, we aim for this work to have laid out a roadmap for multicenter data sharing. Lastly, we have initiated ICUdata as a follow-up project. In this collaboration, we aim to collect and combine data from all ICU patients from as many ICUs as possible in the Netherlands. More information can be found on ICUdata.nl.

The DDW also comes with limitations. First of all, patient transfers could introduce bias since outcomes or prior admission data may not be available for these patients. However, whenever data were available from the receiving hospital, their admissions were connected in the DDW. Moreover, transfers show similar patient characteristics compared to non-transfers upon admission. Therefore, we believe the bias in these data will be limited. Secondly, since ICUs were operating at full capacity at times, it cannot be excluded that some patients that would have been admitted pre-COVID-19 are not currently in this dataset. Thirdly, like any EHR dataset, there will be missing data. We believe that transparency is essential to gauge potential limitations in specific research questions. More importantly, we aspire transparency to lead to changes in clinical practice to improve EHR datasets. Comorbidity data, for example, are frequently not structurally stored in EHRs. We included comorbidity data form Dutch national registry data, which may not be available in other countries. We encourage the community to think about minimally required datasets to be recorded and standardization of EHR parameters. This way, the field of medical data science can advance for the benefit of critically ill patients.

## Conclusion

To the best of our knowledge, the Dutch Data Warehouse is the first dedicated multicenter and full-admission electronic health record database with highly granular clinical data from critically ill COVID-19 patients. We describe solutions for the legal aspects, ETL pipeline, data mapping, data enrichment, and data validation. Currently, 3463 patients are included in the DDW with over 200 million data points from patient demographics, clinical observations, administered medication, laboratory determinations, and vital sign monitors and life support devices. The resulting data warehouse is available to clinicians and researchers within ethical and legal boundaries. We expect this work will encourage clinicians and researchers to be involved in EHR data sharing collaborations to advance the field of medical data science.

## Supplementary Information

**Additional file 1.** Data sharing agreement.

**Additional file 2.** Institutional review board documentation.

**Additional file 3: Table S1.** Overview of derived clinical score.

## Data Availability

All participating hospitals have access to the data. External researchers can get access to all data in collaboration with any of the participating hospitals. The list of collaborators is available in the co-author list and in the declarations section, through the corresponding author, and through the contact details on amsterdammedicaldatascience.nl. Research questions have to be in line with the DSA; to investigate the course of COVID-19 in the ICU and to research potential treatments. Researchers have sign a code of conduct before accessing the data.

## Abbreviations

APACHE II: Acute Physiology and Chronic Health Evaluation II
ALAT: Alanine transferase
API: Application programming interface
ATC: Anatomical Therapeutic Chemical
BMI: Body mass index
DDW: Dutch Data Warehouse
DPO: Data protection officers
DSA: Data sharing agreement
EHR: Electronic health record
ETL: Extract–transform–load
GDPR: General Data Protection Regulation
ICD-10: International Classification of Diseases
IQR: Interquartile range
LOINC: Logical Observation Identifiers Names and Codes
RASS: Richmond Agitation and Sedation Scales
SNOMED-CT: Systematized Nomenclature of Medicine Clinical Terms
SOFA: Sequential Organ Failure Assessment
SQL: Structured Query Language
