# Supplementary material for: The Dutch Data Warehouse, a multicenter and full-admission electronic health records database for critically ill COVID-19 patients
Source: Crit Care. 2021 Aug 23;25:304. doi: 10.1186/s13054-021-03733-z (PMC8381710; doi:10.1186/s13054-021-03733-z)
Supplement: Supplementary file 1 — Additional file 1. Data sharing agreement. [file 13054_2021_3733_MOESM1_ESM.pdf]

AGREEMENT ON THE SHARING OF PSEUDONYMIZED PERSONAL DATA
(for research)

This agreement (hereinafter referred to as “Agreement”) is made and entered by and between:

____

legally represented by the undersigned, hereinafter referred to as “PROVIDER”

and

____

PROVIDER and RECIPIENT hereinafter jointly referred to as “Parties” and individually as “Party”;

WHEREAS

a) PROVIDER has obtained and / or generated DATA as further defined below;
b) RECIPIENT, through _____________________, hereinafter referred to as “RECIPIENT SCIENTIST”, has requested PROVIDER, through ______________________, hereinafter referred to as “PROVIDER'S SCIENTIST”, to provide RECIPIENT with the DATA for use by RECIPIENT’S SCIENTIST for the purpose of its RECIPIENT’S RESEARCH PLAN;
c) The purpose and means of RECIPIENT’S RESEARCH PLAN have been determined by RECIPIENT;
d) PROVIDER is willing, subject to the terms and conditions of this Agreement, to provide the DATA to RECIPIENT.

I Definitions

1. DATA: the data being transferred under this Agreement is the data that is further specified in Annex I to this Agreement, provided without directly identifying personal information. The DATA constitutes pseudonymized personal health data under the GDPR.
2. RECIPIENT’S RESEARCH PLAN: The research plan specified in Annex II to this Agreement for which the DATA may be used.
3. EFFECTIVE DATE: The date of last signing of this Agreement.
4. INVENTION: any invention, discovery , improvement, material, signal, process, formula, know-how or other innovation related to or arising from the use of the DATA and/or CONFIDENTIAL INFORMATION , whether patentable or not and obtained as a result of the performance of RECIPIENT’S RESEARCH PLAN.
5. CONFIDENTIAL INFORMATION: All information, know-how, grant applications, method of work, techniques, expertise of PROVIDER regarding the DATA, its characteristics and PROVIDER’s research concerning the DATA, whether of a scientific, technical, engineering, operational, or economic nature, supplied to or obtained by RECIPIENT in written form, in the form of drawings or in the recording of oral conversation, or samples.
6. GDPR: the Regulation (EU) 2016/679 of the European Parliament and of the Council of 27 April 2016 on the protection of natural persons with regard to the processing of personal data and on the free movement of such data (General Data Protection Regulation)
7. APPLICABLE DATA PROTECTION LAW*:* the GDPR and any additional locally applicable data protection legislation.

8. SUBJECT(S): shall mean the patient or other person from whom the DATA was obtained.

II. Terms and Conditions of this Agreement:

1. The DATA and any other information provided is made available as a service to the research community and no ownership rights in the DATA and any other information shall be obtained by RECIPIENT under this Agreement.

2. a) DATA shall be provided by PROVIDER in a sufficiently secure manner and Parties shall handle all DATA in accordance with the APPLICABLE DATA PROTECTION LAW and shall keep such DATA confidential without any of the exclusions contained in Article 11 below.
b) With respect to the DATA, RECIPIENT shall be considered to be a separate data controller under the APPLICABLE DATA PROTECTION LAW for the processing of the DATA for RECIPIENT'S RESEARCH PLAN.
c) RECIPIENT shall implement appropriate technical and organizational measures to meet the requirements for data controllers of the APPLICABLE DATA PROTECTION LAW.
d) If RECIPIENT becomes aware of a personal data breach, RECIPIENT shall promptly notify PROVIDER. In such a case Parties will fully cooperate with each other to remedy the personal data breach, fulfill the statutory notification obligations timely and cure any damages. The term 'personal data breach' refers to articles 33 and 34 of GDPR.
e) In the event that SUBJECT withdraws his/her permission for the use thereof, PROVIDER shall supply RECIPIENT with sufficient information and RECIPIENT shall immediately cease all use of the relevant DATA and shall delete all copies of the relevant DATA. Upon request from PROVIDER, RECIPIENT shall confirm in writing the complete deletion of such DATA.
f) PROVIDER shall be data controller of the DATA under the GDPR up until the moment the DATA is provided to RECIPIENT.

The Parties' contact details for inquiries regarding handling and protection of DATA are as follows:

For RECIPIENT, to:

Name:
Tel:
E-mail:

For PROVIDER, to:

Name:
Address:
e-mail:

3. RECIPIENT shall not attempt in any way to obtain the identity of the SUBJECTS. The RECIPIENT and the RECIPIENT SCIENTIST agree that the DATA: (a) is to be used only for the academic purposes as described in RECIPIENT'S RESEARCH PLAN; (b) will not be used for other, including commercial purposes. Furthermore, in carrying out the RECIPIENT'S RESEARCH PLAN, RECIPIENT shall not allow third parties that are not expressly mentioned in the Annexes to access or otherwise process the DATA without prior written approval of PROVIDER. However, as an exception to the foregoing, such prior approval shall not be required for service providers in the context of the standard business operations of PROVIDER, such as parties who supply ICT infrastructure maintenance. RECIPIENT will

safeguard that any data processors who have access to the DATA are instructed by a binding agreement to process the personal data in accordance with the requirements stated in the GDPR.

Notwithstanding the restrictions on transfer given directly above, RECIPIENT may transfer the DATA to E.E.R. based third parties solely for the purpose of the RECIPIENT'S RESEARCH PROJECT. Such transfers shall only be made by RECIPIENT after the conclusion of a written agreement with terms at least as strict as the terms of this Agreement and without any further right of transfer.

4. Upon request, RECIPIENT'S SCIENTIST shall keep PROVIDER'S SCIENTIST informed of the results arising from RECIPIENT'S RESEARCH PLAN.

5. RECIPIENT will report any INVENTIONS to PROVIDER and PROVIDER'S SCIENTIST. RECIPIENT shall promptly provide PROVIDER with a detailed written description of the INVENTION and indicate the role, if any, of any of RECIPIENT's employees in creating the INVENTION. Ownership of INVENTIONS will be joint ownership. Both Parties shall make appropriate mutual arrangements concerning the protection and exploitation of such joint INVENTION. Until such agreement is effective, each Party shall be entitled to use the joint INVENTION for research purposes, but neither Party shall be entitled to exploit, disclose, license or transfer its rights in connection with the joint INVENTION.

6. Except as provided in this agreement, no express or implied licenses or other rights are provided to the RECIPIENT under any Intellectual Property (IP) rights of PROVIDER.

7. The DATA will be provided at no cost or with an optional transmittal fee solely to reimburse PROVIDER for the collection and/or preparation of the DATA. No fee is requested.

8. DATA will be provided to the RECIPIENT by PROVIDER's SCIENTIST in a sufficiently secure manner and in a format to be agreed upon by the RECIPIENT SCIENTIST and the PROVIDER's SCIENTIST.

9. PROVIDER warrants a) that it has verified that there is an appropriate legal ground for the provision of the DATA to RECIPIENT in accordance with the GDPR (such as Article 6 and/or 5.1 sub b GDPR) b) that there is a valid exception to the prohibition for processing personal health data (Article 9 GDPR) and c) that it shall be provided under approval from the relevant ethics committee to the extent required. Apart from this, it is expressly understood that PROVIDER does not make any warranties regarding the DATA and specifically does not warrant or guarantee that the DATA will be accurate, be merchantable or useful for any particular purpose. PROVIDER cannot and shall not be held liable for any claims or damages by RECIPIENT or any third party, in connection with or as a result of the use of DATA by RECIPIENT. Unless and to the extent caused by PROVIDER's gross negligence or willful misconduct, RECIPIENT undertakes to hold harmless PROVIDER at all times against all of such damages or claims.

In regards to the DATA and personal data breaches, RECIPIENT shall be responsible and liable for any damages, losses and fines resulting from its own actions or failures to adhere to the terms of this Agreement and APPLICABLE DATA PROTECTION LAW and RECIPIENT shall indemnify and hold harmless PROVIDER for any of such damages. For the purposes of this sub clause, actions or omissions of data processors contracted by RECIPIENT, shall be attributed to RECIPIENT.

10. RECIPIENT agrees in its use of the DATA to comply with all applicable international and national laws, statutes, regulations and guidelines.

11. RECIPIENT shall treat all CONFIDENTIAL INFORMATION as confidential for the duration of this Agreement including any extension thereof and thereafter for a period of five (5) years following termination or expiry of this Agreement. Excluded from this obligation of confidentiality shall be any CONFIDENTIAL INFORMATION of which the RECIPIENT can reasonably demonstrate that it (a) was previously known to RECIPIENT, or (b) is, and/or becomes, publicly available during said five (5) year period through no fault of RECIPIENT, or (c) is independently and lawfully developed by the RECIPIENT, or (d) was published or otherwise disseminated in accordance with the publication procedure set out below in article 12. However, the foregoing exceptions shall not apply to: (a) CONFIDENTIAL INFORMATION contained within more general information that may fall within one or more of the exceptions, or (b) any combination of features or items of CONFIDENTIAL INFORMATION where one or more of the relevant individual features or items (but not the combination itself) may fall within one or more of the exceptions. The obligation of confidentiality shall not apply to any disclosure required by law, provided that RECIPIENT shall notify PROVIDER of any disclosure required by law in sufficient time so that PROVIDER may contest such requirement, if PROVIDER so chooses.

12. Parties acknowledge the importance of disseminating the results of the RECIPIENT'S RESEARCH PROJECT. Therefore, RECIPIENT shall endeavor to publish or otherwise publicly disclose information, any data, results or information generated using the DATA ("Disclosure(s)"), after review by PROVIDER. The following shall apply to Disclosures:
a) Authorship of any publications shall follow the principles set out in the ICMJE recommendations 'Defining the Role of Authors and Contributors' as can be found on http://www.icmje.org/recommendations/browse/roles-and-responsibilities/defining-the-role-of-authors-and-contributors.html .
b) At least 30 days before RECIPIENT submits a paper or abstract for Disclosure, RECIPIENT shall provide such paper or abstract to PROVIDER, who will have 30 days to review proposed manuscripts and 15 days to review proposed abstracts to assure that its CONFIDENTIAL INFORMATION is protected. It is agreed that RECIPIENT will fully comply with any reasonable written request by PROVIDER to omit specified CONFIDENTIAL INFORMATION of PROVIDER from such paper, abstract, press release or other disclosure prior to Disclosure.
c) During the acute COVID-19 crisis situation speedy publication of relevant findings is needed. Article 12 c will replace article 12 b until 31 December January 2021 or earlier if the COVID-19 crisis is ended. At least 3 days before RECIPIENT publish a paper or abstract for Disclosure, RECIPIENT shall provide such paper or abstract to PROVIDER, who will have 3 days to review submitted manuscripts to assure that its CONFIDENTIAL INFORMATION is protected. It is agreed that RECIPIENT will fully comply with any reasonable written request by PROVIDER to omit specified CONFIDENTIAL INFORMATION of PROVIDER from such paper, abstract, press release or other disclosure prior to publication

d) In every Disclosure by RECIPIENT based upon results obtained from the research through the help of the received DATA provided by PROVIDER, RECIPIENT shall appropriately acknowledge PROVIDER and PROVIDER'S SCIENTIST as contributor of the DATA.

13. This Agreement will become effective on the Effective Date and will terminate 5 years after the Effective Date, unless mutually extended in writing by both Parties. Any clauses which will be expected or intended by its nature to survive the termination or the expiration of this Agreement, shall survive the termination or the expiration of this Agreement.

Upon expiration or termination of this Agreement, the right to use the DATA and CONFIDENTIAL INFORMATION will automatically end. After expiration or termination of this Agreement, RECIPIENT will destroy all DATA received from PROVIDER. Upon request from PROVIDER, RECIPIENT shall confirm in writing the complete deletion of such DATA and CONFIDENTIAL INFORMATION. However, RECIPIENT may retain one copy of the DATA solely to comply with DATA transfer requests that third party academic institutions may make for replication of the RECIPIENT'S RESEARCH PROJECT, as described in Article 3 above.

14. This Agreement will be construed, governed, interpreted and enforced according to the laws of the Netherlands. Parties will first strive to settle any disputes amicably before taking legal action. All disputes arising out of or in relation to this Agreement that cannot be settled amicably will be brought before the competent court in the Netherlands, in the district in which the Provider resides.

15. This Agreement will be binding upon and inure to the benefit of the respective successors and assignees of the Parties hereto. However, RECIPIENT may not assign this Agreement in whole or in part without the prior written consent of the PROVIDER.

16. This Agreement may only be altered or amended by an instrument in writing signed by all of the Parties.

17. If any portion of this Agreement is in violation of any applicable regulation, or is unenforceable or void for any reason whatsoever, such portion will be inoperative and the remainder of this Agreement will be binding upon the Parties.

18. Both Parties acknowledge that the signatories to this Agreement are authorized representatives of each of the Parties and legally authorized to sign this Agreement.

19. If the lawful performance of any part of this Agreement by a Party is rendered impossible by or as a result of any cause beyond such Party's reasonable control, such Party will not be considered in breach hereof as a result of failing so to perform.

IN WITNESS WHEREOF, the Parties have executed this Agreement, in duplicate originals or as a signed PDF, as of the Effective Date.

For the **PROVIDER**	For **RECIPIENT**,
By:____	By:____
Name:	Name:
Title	Title
Date:	Date:

**READ AND ACKNOWLEDGED:**	**READ AND ACKNOWLEDGED:**
_____________________________________	_____________________________________
PROVIDER'S SCIENTIST	RECIPIENT'S SCIENTIST
