# Supplementary material for: The Dutch Data Warehouse, a multicenter and full-admission electronic health records database for critically ill COVID-19 patients
Source: Crit Care. 2021 Aug 23;25:304. doi: 10.1186/s13054-021-03733-z (PMC8381710; doi:10.1186/s13054-021-03733-z)
Supplement: Supplementary file 2 — Additional file 2. Institutional review board documentation. [file 13054_2021_3733_MOESM2_ESM.pdf]

**Please note: all names from individuals and organizations have been removed.**

# Onderzoeksprotocol

**(voor aanvraag niet-WMO verklaring)**

## Algemene gegevens

| | |
|---|---|
| **Titel** | Machine learning voor de behandeling van COVID-19 patiënten op de intensive care |
| **Datum** | 18-03-2020 |
| **Versienummer** | 1 |
| **Indiener** | |
| **Coördinerende onderzoeker** | |
| **Hoofdonderzoeker(s)** | |
| **Opdrachtgever (verrichter)** | |

## Onderzoekgegevens

| | |
|---|---|
| **Rationale** | Nederland gaat gebukt onder de Corona Crisis. De ziekenhuiszorg is in opperste staat van paraatheid. Het openbare leven is tot stilstand gekomen door de drastische maatregelen om de verspreiding van het severe acute respiratory syndrome coronavirus 2 (SARS-CoV-2) te beteugelen. Het is de verwachting dat meer dan de helft van de Nederlanders uiteindelijk besmet raakt. En dat betekent ook dat een groot aantal patiënten met coronavirus disease 2019 (COVID-19) op de intensive care behandeld zal worden.<br><br>Over welke combinatie van behandelstrategieën bij een individuele intensive care patiënt met COVID-19 leidt tot het optimale resultaat is weinig tot niets bekend. Waarschijnlijk zal de behandeling daarom per |

| | |
|---|---|
| | ziekenhuis en per intensivist verschillen, waarbij er voor verschillende combinaties van behandelstrategieën gekozen wordt, de zogenaamde praktijkvariatie.<br><br>Tegelijkertijd bestaat op de intensive care de unieke situatie dat van iedere opgenomen patiënt grote hoeveelheden gegevens routinematig worden vastgelegd in het elektronisch patiëntendossier. Het gaat hierbij om gegevens uit apparatuur voor bewaking en ondersteuning van vitale functies (bijvoorbeeld bloeddruk, pols, SpO2, ademfrequentie, beademingsinstellingen), laboratoriumdata (bijvoorbeeld CRP, leukocyten), ook observationele data (bijvoorbeeld urineproductie), en data over alle behandelstrategieën.<br><br>Met behulp van deze grote hoeveelheden routinematig opgeslagen gegevens, zou machine learning in staat moeten zijn om te leren welke combinatie van behandelstrategieën bij een individuele patiënt geassocieerd is met het beste resultaat. |
| **Doel** | Analyseren welke combinatie van behandelstrategieën op de intensive care bij individuele patiënt met COVID-19 geassocieerd is met de beste uitkomsten. |
| **Studie design** | Gegevensverzameling van COVID-19 patiënten die behandeld zijn of worden op afdelingen intensive care volwassenen in Nederlandse ziekenhuizen. Hierbij worden in ieder geval demografische data, gegevens over vitale parameters, lab data, en observationele data verzameld. Met behulp van machine learning technieken wordt deze data geanalyseerd, |
| **Studie populatie** | Patiënten opgenomen op intensive care afdelingen. Volwassenen, leeftijd >= 18 jaar |
| **Inclusiecriteria** | Alle patiënten opgenomen op een intensive care volwassenen die positief getest zijn voor het severe acute respiratory syndrome coronavirus 2 (SARS-CoV-2) |
| **Exclusiecriteria** | Geen |

| | |
|---|---|
| **Aantal proefpersonen/ sample grootte** | Dit zal afhankelijk zijn van de grootte van de COVID-19 patiënten die uiteindelijk op de intensive care terechtkomen. |
| **Werving proefpersonen** | Patiënten zullen geselecteerd worden op basis van een positieve testuitslag voor SARS-CoV-2 |
| **Interventie** | Niet van toepassing |
| **Standaardzorg / Standaardbehandeling** | Standaardzorg zal de zorg omvatten die gegeven wordt op de verschillende intensive care units. Op dit moment is er weinig bewijs voor de behandeling van deze patiëntengroep, waardoor we verwachten dat er een variatie is aan behandeling. |
| **Studie parameters** | Alle verzamelde gegevens zullen worden meegenomen indien deze relevant blijken voor het model. |
| **Studie eindpunten** | Accuraatheid en precisie van het te ontwikkelen model dat gebruikt kan worden om de behandelkeuzes te identificeren die geassocieerd zijn met een betere uitkomst van patiënten |
| **Statistische analyses** | Modellering op basis van machine learning technieken. De ontwikkeling van de algoritmen en analyse van de resultaten zal mede uitgevoerd worden door ***, in Python. De baseline karakteristieken van patiënten en de uitkomsten van de modellen worden geanalyseerd middels standaard descriptieve en vergelijkende statistiek. |
| **Belasting voor de proefpersoon** | Er is geen directe belasting voor de proefpersonen |
| **Risico voor de proefpersoon** | Er is geen direct risico voor de proefpersoon omdat er geen interventies plaatsvinden. |
| **Voordelen deelname aan het onderzoek** | Niet van toepassing |
| **Nadelen deelname aan het onderzoek** | Niet van toepassing |
| **Vergoeding voor de proefpersoon** | Niet van toepassing |
| **Administratieve aspecten** | De data zal gecodeerd aangeleverd worden |
| **Publicatiebeleid en amendementen** | Conform het vigerende publicatiebeleid, waarbij zowel positieve als negatieve resultaten ter publicatie worden aangeboden. |

| | |
|---|---|
| **Overige punten van belang voor de METc** | |

**Please note: all names from individuals and organizations have been removed.**

# Resultaat Privacy Impact Assessment (PIA)

Project : Machine learning voor de behandeling van COVID-19 patiënten op de intensive care

Datum PIA: 18-03-2020

Uitvoerenden:

| Naam | Functie | Afdeling |
|---|---|---|

Versie: 1 datum 18-03-2020

| # | Vraag | J/N | Toelichting / informatie |
|---|---|---|---|
| 1 | Het type project | | |
| 1.1 | Is er sprake van het verwerken van persoonsgegevens? | J | Gegevens van coronavirus disease 2019 (COVID-19) patiënten die opgenomen zijn op intensive care units in Nederland<br>Vitale parameters, lab gegevens, demografische informatie, medicatiegegevens. Gegevens betreffende NAW, geboortemaand en dag worden niet in de dataset opgenomen. |
| 1.2 | Is het duidelijk wie verantwoordelijk is voor de verwerking van de gegevens? | J | Verantwoordelijk =<br>Projectleider:<br>Uitvoerend: |

| # | Vraag | J/N | Toelichting / informatie |
|---|---|---|---|
| 1.3 | Verwerkt uw organisatie de persoonsgegevens in opdracht en onder verantwoordelijkheid van een andere organisatie? Ofwel: Treedt uw organisatie op als bewerker? | N | |
| 1.4 | Is het duidelijk wie na afloop van het project verantwoordelijk is voor het in stand houden en evalueren van de getroffen maatregelen? | J | |
| 1.5 | Is het doel van de verwerking van persoonsgegevens binnen het project voldoende SMART omschreven? | J | Ja. Het doel is het analyseren welke combinatie van behandelstrategieën bij COVID-19 patiënten geassocieerd zijn met de beste uitkomsten, zodat patiënten met COVID-19 optimaal behandeld kunnen worden. Onderzoeksprotocol C1 versie 1 bijgevoegd. |
| 1.6 | Is er sprake van: | | |
| a. | Gebruik van nieuwe technologie? | N | Gebruik makende van machine learning |
| b. | Gebruik van technologie die bij het publiek vragen of weerstand op kan roepen? | J | Risico: onbegrip bij publiek over het inzetten van machine learning in de zorg.<br>Beheersmaatregel: middels externe communicatie bekendmaken van onderzoek en resultaten. Tevens wordt in de communicatie bezwaarmogelijkheden voor patiënten bekendgemaakt in het kader van het transparantiebeginsel |
| c. | De invoering van bestaande technologie in een nieuwe context? | N | |
| d. | (Andere) grote verschuivingen in de werkwijze van de organisatie, de manier waarop persoonsgegevens worden verwerkt en/of de technologie die daarbij gebruikt wordt? | J | Het gaat over de inzet van machine learning, waarbij alle klinische data van COVID-19 patienten op de intensive care gebruikt zal worden |

| # | Vraag | J/N | Toelichting / informatie |
|---|---|---|---|
| e. | Een nieuwe verwerking van persoonsgegevens | J | |
| f. | Het verzamelen van meer of andere persoonsgegevens dan voorheen of een nieuwe manier van verzamelen. | N | Het betreft analyses van bestaande gegevens uit de bronsystemen van intensive care afdelingen |
| g. | Gebruik van al verzamelde gegevens voor een nieuw doel of een nieuwe manier van gebruiken. | N | Doel is inzicht geven in de optimale therapie voor COVID-19 patiënten op de intensive care |
| 1.7 | Heeft u op alle bovenstaande (a t/m g) **nee** geantwoord? | N | |
| 1.8 | Is er (naast de Avg) veel wet- en regelgeving ten aanzien van persoonsgegevens waar het project mee te maken heeft? | J | WGBO, BIG, WKKGZ en WMO |
| 1.9 | Zijn er veel maatschappelijke belanghebbenden? | J | COVID-19 patiënten die opgenomen zijn of worden op intensive care units. Alle daarbij betrokken gezondheidszorg organisaties |
| 1.10 | Zijn er veel partijen betrokken bij de uitvoering van het project? | J | Intensive care units waar COVID-19 patiënten behandeld worden. |
| 1.11 | Is er een geschillenregeling of een partij waar de betrokkene terecht kan bij vragen of klachten? | J | Standaard geschillenregeling |
| | | | |
| 2 | De gegevens | | |

| # | Vraag | J/N | Toelichting / informatie |
|---|---|---|---|
| 2.1 | Zijn alle gegevens nodig om het doel te bereiken (worden er zo min mogelijk gegevens verzameld)? | J | Machine learning kenmerkt zich door het gebruik van een integrale dataset van COVID-19 patiënten op de intensive care |
| 2.2 | Kan het doel met geanonimiseerde of gepseudonimiseerde gegevens worden bereikt (terwijl daar op dit moment geen gebruik van wordt gemaakt)? | J | Het betreft gespseudonimiseerde gegevens. Het sleutelbeheer is in beheer van de aanleverende ziekenhuizen. |
| 2.3 | Kunnen de gegevens gebruikt worden om het gedrag, de aanwezigheid of prestaties van mensen in kaart te brengen en/of te beoordelen (ook al is dit niet het doel)? | N | |
| 2.4 | Is er sprake van het verwerken van: | | |
| a. | Bijzondere persoonsgegevens? | J | Medische gegevens intensive care |
| b. | Uniek identificerende gegevens? | J | Gepseudonimiseerde patientennummers van betreffende ziekenhuis. In verband met mogelijke heropname is identificatie op basis van een gepseudonimiseerd patiëntennummer noodzakelijk |
| c. | Wettelijk voorgeschreven persoonsnummers. | N | |
| d. | Andere gegevens dan hiervoor beschreven waarvoor geldt dat sprake is van een (gepercipieerde) verhoogde gevoeligheid? | N | |

| # | Vraag | J/N | Toelichting / informatie |
|---|---|---|---|
| 2.4.1 | Bij een van bovenstaande Ja: kan het doel met andere gegevens worden bereikt die een verminderd risico op misbruik met zich mee brengen? | N | |
| 2.5 | Verwerkt u gegevens over kwetsbare groepen of personen? | J | Het betreft COVID-19 patiënten van de intensive care |
| 2.6 | Hebben de gegevens betrekking op de gehele of grote delen van de bevolking? | N | Het betreft COVID-19 patiënten op de intensive care |
| | | | |
| 3 | Betrokken partijen | | |
| 3.1 | Zijn er (na afronding van het project) bij het verzamelen en verder verwerken van de gegevens meerdere interne partijen betrokken? | N | Afdeling intensive care |
| 3.2 | Zijn er (na afronding van het project) bij het verzamelen en verder verwerken van de gegevens meerdere externe partijen betrokken? | J | |
| 3.3 | Zijn er partijen betrokken (in het project of bij de verwerking) die zich niet aan een met Nederland vergelijkbare privacywetgeving hoeven te houden? | N | |

| # | Vraag | J/N | Toelichting / informatie |
|---|---|---|---|
| 3.4 | Is de verstrekking van de gegevens aan derde partijen in lijn met het doel waarvoor de gegevens oorspronkelijk zijn verzameld? | J | Dit is verenigbaar met de uitzonderingen voor medisch wetenschappelijk onderzoek |
| 3.5 | Worden de gegevens verkocht aan derde partijen? | N | |
| | | | |
| 4 | Verzamelen van gegevens | | |
| 4.1 | Kan de manier waarop de gegevens worden verzameld worden opgevat als privacy gevoelig? | N | Gegevens komen uit intensive care systemen en EPD |
| 4.2 | Is het doel van het verzamelen van de gegevens publiekelijk bekend of kan het publiekelijk bekend gemaakt worden? | J | Risico: onvoldoende transparantie over het project<br>Beheersmaatregel: separate communicatie over het project en het doel van het project en de rechten van betrokkenen (recht op bezwaar). |
| 4.3 | Verzamelt u de gegevens op basis van een van de wettelijke grondslagen? | J | WGBO |
| 4.4 | Is duidelijk of u de gegevens verzamelt op basis van opt-in (verzameling uitsluitend als de betrokkene daarvoor toestemming heeft gegeven) of op basis van opt-out (verzameling tenzij de betrokkene daartegen bezwaar heeft gemaakt)? | Nvt | |

| # | Vraag | J/N | Toelichting / informatie |
|---|---|---|---|
| 4.4.1 | Indien u toestemming aan de betrokkene vraagt (opt-in), kunnen de betrokkenen de toestemming op een later tijdstip intrekken (opt-out)? | Nvt | |
| 4.4.2 | Is de impact van het intrekken van de toestemming groot voor de betrokkene? | Nvt | |
| 4.5 | Meldt u de betrokkene dat de gegevens worden verzameld? | J | Privacystatement en separate berichtgeving over project. Tevens privacystatement betrokken ziekenhuizen |
| 4.5.2 | Bij Ja (op vraag 4.5): meldt u de betrokkene waarom de gegevens worden verzameld (wat u er mee gaat doen)? | J | Privacystatement en separate berichtgeving over project. Tevens privacystatement betrokken ziekenhuizen |
| 4.5.3 | Bij Ja: (op vraag 4.5): meldt u de betrokkene aan wie de gegevens worden verstrekt (daar waar dit geen wettelijke verplichting is)? | J | Privacystatement en separate berichtgeving over project. Tevens privacystatement betrokken ziekenhuizen |
| 4.6 | Zou de betrokkene kunnen worden verrast door de verwerking (op het moment dat hij daarover wordt geïnformeerd)? | N | |
| | | | |
| 5 | Gebruik van gegevens | | |
| 5.1 | Is het gebruik van de gegevens verenigbaar (in lijn) met het doel van het verzamelen? | J | |

| # | Vraag | J/N | Toelichting / informatie |
|---|---|---|---|
| 5.2 | Worden de gegevens gebruikt voor andere bedrijfsprocessen of doelen dan waar ze oorspronkelijk voor verzameld zijn? | N | |
| 5.3 | Is de kwaliteit van de gegevens gewaarborgd, dat wil zeggen: zijn de gegevens actueel, juist en volledig? | J | Gegevens komen rechtstreeks uit bronsystemen van intensive care units in betreffende ziekenhuizen |
| 5.4 | Worden op basis van de gegevens beslissingen genomen over de betrokkenen? | N | Ten behoeve van de keuze van de behandeling van COVID-19 toekomstige intensive care patiënten |
| 5.5 | Is sprake van koppeling, verrijking of vergelijking van gegevens uit verschillende bronnen? | J | Het betreft gegevens van verschillende intensive care units |
| 5.6 | Worden de gegevens breed verspreid binnen de organisatie? | N | |
| 5.7 | Worden de gegevens verspreid buiten de organisatie? | J | Gegevens zijn opvraagbaar door aanleverende instellingen |
| 5.8 | Stelt uw organisatie profielen op van de betrokkenen, al dan niet geanonimiseerd? | N | |
| 5.9 | Kunnen de betrokkenen hun gegevens inzien of daarom vragen? | J | Op aanvraag via standaardprocedures en betreffende ziekenhuis waar patiënt gelegen heeft |
| 5.10 | Kunnen de betrokkenen hun gegevens corrigeren of daarom vragen (verbeteren, aanvullen)? | J | Op aanvraag via standaardprocedures en betreffende ziekenhuis waar patiënt gelegen heeft |
| 5.11 | Kunnen de betrokkenen hun gegevens verwijderen of daarom vragen? | J | Na verzoek worden gegevens uit de bronsystemen verwijderd |

| # | Vraag | J/N | Toelichting / informatie |
|---|---|---|---|
| | Dataportabiliteit | | |
| 5.12 | Indien u de persoonsgegevens van betrokkene digitaal verwerkt en de u gegevens op basis van toestemming van betrokkenen of een overeenkomst met betrokkene heeft verkregen, bent u in staat om de gegevens digitaal aan betrokkene ter beschikking te stellen dan wel betrokkene te faciliteren om de gegevens digitaal aan een andere verantwoordelijke ter beschikking te stellen?<br><br>Het gaat om de gegevens die u van de betrokkenen heeft ontvangen of bij de betrokkenen heeft geobserveerd. | Nvt | |
| | Recht op beperking (Nieuw AVG recht) | | |
| 5.13 | Kan de verwerking van de gegevens van betrokkene gedurende een bepaalde periode worden beperkt indien betrokkene daarom verzoekt? | Nvt | . |
| 6 | Bewaren en vernietigen | | |
| 6.1 | Is een bewaartermijn voor de gegevens vastgesteld? | J | Conform wetenschappelijk onderzoek wordt de wettelijke termijn voor bewaren onderzoeksgegevens aangehouden |

| # | Vraag | J/N | Toelichting / informatie |
|---|---|---|---|
| 6.2 | Kunnen de gegevens na afloop van de bewaartermijn fysiek worden verwijderd (uit een bestand) of vernietigd (papier)? | J | Binnen technische mogelijkheden |
| 6.2.1 | Zo ja (op vraag 6.2), worden de gegevens na verstrijken van de bewaartermijn op zo'n manier vernietigd of verwijderd dat ze niet meer te benaderen en te gebruiken zijn? | J | Binnen technische mogelijkheden |
| | | | |
| 7 | Beveiliging | | |
| 7.1 | Is sprake van intern geformuleerd beleid over het beveiligen van informatie? | J | Beleid informatiebeveiliging, privacybeleid en identity en access management |
| 7.2 | Zo ja (op vraag 7.1), is duidelijk met welke maatregelen er voor wordt gezorgd dat aan de gestelde eisen in het beveiligingsbeleid voldaan gaat worden? | J | NEN7510 |
| | | | |
| 8 | Meldplicht datalekken | | |
| 8.1 | Zijn maatregelen getroffen om datalekken indien noodzakelijk te melden aan de Autoriteit persoonsgegevens en aan de getroffen personen van wie de gegevens zijn gelekt? | J | Standaard procedure voor signaleren en afhandelen van datalekken |

| # | Vraag | J/N | Toelichting / informatie |
|---|---|---|---|
| 8.2 | Zo ja, is bij het vaststellen van de maatregelen rekening gehouden met de Richtsnoeren die de Autoriteit persoonsgegevens over de meldplicht datalekken heeft gepubliceerd ? | J | |
| | | | |

Geven de uitkomsten van de analyse aanleiding om dit project niet uit te voeren?

Welke risico's zijn er geïdentificeerd?

Is het risico aanvaardbaar ?

Zo nee welke maatregelen worden genomen om de risico te beperken tot een aanvaardbaar niveau

| Risico | Aanvaardbaar J/N | Maatregelen | Status |
|---|---|---|---|
| Onvoldoende beveiligingsniveau externe verwerker | N | Verwerkingsovereenkomst BOZ | Reeds gerealiseerd |
| Onvoldoende transparantie | N | Via separate berichtgeving zullen betrokken geïnformeerd worden over het doel van de verwerking van de gegevens en de rechten van betrokken | Bij aanvang project |

Meldingsnummer bij het centraal meldpunt gegevensverwerking(CMG nummer) van deze verwerking:

Een CMG nummer is te verkrijgen via het meldingsformulier
