# Supplementary material for: The Dutch Data Warehouse, a multicenter and full-admission electronic health records database for critically ill COVID-19 patients
Source: Crit Care. 2021 Aug 23;25:304. doi: 10.1186/s13054-021-03733-z (PMC8381710; doi:10.1186/s13054-021-03733-z)
Supplement: Supplementary file 3 — Additional file 3: Table S1. Overview of derived clinical score. [file 13054_2021_3733_MOESM3_ESM.docx]

## Additional files

**Additional Table 1.** Overview of derived clinical scores

| **Clinical score** | **Description** |
| --- | --- |
| --- | --- |
| adjusted_sofa_partial | Sum of all non-null SOFA subscores. |
| adjusted_sofa_total | Sum of all SOFA subscores. It's `null` if _any_ of the subscores is `null`. |
| aki | AKI stage using the creatinine and renal replacement therapy criteria. |
| aki | AKI stage using the urine criteria. |
| apache_score_partial | Sum of all non-null APACHE subscores. |
| apache_score | Sum of all APACHE subscores. It's `null` if _any_ of the subscores is `null`. |
| rox_index | `o2_saturation` divided by `fio2` divided by `respiratory_rate_measured`. Only measured at the time when optiflow/high flow is started. |
| rox_index | `o2_saturation` divided by `fio2` divided by `respiratory_rate_measured`. Only calculated if the patient is under optiflow/high flow. |

aki: acute kidney injury
